# First UK patient cohort treated with stereotactic ablative radiotherapy for primary kidney cancer

**DOI:** 10.1002/bco2.199

**Published:** 2023-04-13

**Authors:** Anjali Zarkar, Dan Henderson, Antony Carver, Geoff Heyes, Victoria Harrop, Sarah Tutill, Julie Kilkenny, Andrea Marshall, Nada Elbeltagi, Helen Howard

**Affiliations:** ^1^ University Hospitals Birmingham NHS Foundation Trust Birmingham UK; ^2^ Warwick Clinical Trials Unit Warwick UK

**Keywords:** primary kidney SABR, SABR, SABR for primary RCC, SBRT, SBRT for primary RCC, stereotactic ablative radiotherapy, stereotactic radiotherapy

## Abstract

**Aims:**

Stereotactic ablative radiotherapy (SABR) for primary renal cell carcinoma (RCC) is a promising non‐invasive ablative treatment option. A prospective interventional clinical trial published showed that treatment was feasible and well tolerated. We present the first single‐institution UK cohort of patients with primary RCC receiving protocol‐based SABR with prospective follow‐up. We also present a protocol that could be used to facilitate more widespread use of the treatment.

**Materials and methods:**

Nineteen biopsy‐proven primary RCC patients were treated with either 42 Gy in three fractions on alternate days or 26 Gy in a single fraction based on predefined eligibility criteria using either Linear Accelerator or CyberKnife platform. Prospective toxicity data using CTCAE V4.0 and outcome data such as estimated glomerular filtration rate (eGFR) and tumour response using CT thorax, abdomen and pelvis (CT‐TAP) were collected at 6 weeks, 3, 6, 12, 18 and 24 months post treatment.

**Results:**

The 19 patients had a median age of 76 years (interquartile range [IQR] 64–82 years) and 47.4% were males, and they had a median tumour size of 4.5 cm (IQR 3.8–5.2 cm). Single and fractionated treatment was well tolerated and there were no significant acute side effects. The mean drop from baseline in eGFR at 6 months was 5.4 ml/min and that at 12 months was 8.7 ml/min. The overall local control rate at both 6 and 12 months was 94.4%. Overall survival at 6 and 12 months was 94.7% and 78.3%, respectively. After a median follow‐up of 17 months, three patients experienced a Grade 3 toxicity, which was resolved with conservative management.

**Conclusion:**

SABR for primary RCC is a safe and feasible treatment for medically unfit patients, which can be delivered in most UK cancer centres using standard Linear Accelerator as well as CyberKnife platforms.

## 1 INTRODUCTION

Kidney cancer incidence is increasing with approximately 13 100 new cases per year within the United Kingdom. Accounting for 4% of all cancer cases, kidney cancer is strongly related to age, with the highest incidence rates being in older men and women between 85 and 89 years.^1^ The majority (56%) of patients are diagnosed with localised stage I and II diseases, compared with late stage (stages III and IV) with the 5‐year overall survival rate being 85% for stage 1 disease and 75% for stage II disease.^1^

Renal cell carcinoma (RCC) is traditionally considered to be a radio‐resistant tumour with surgical excision being the current standard of care for primary kidney cancer. However, a significant proportion of patients are considered unsuitable for surgery due to concurrent co‐morbidities and frailty.

Surgically unfit patients with primary RCCs may be offered invasive ablative procedures such as radiofrequency ablation (RFA) or cryoablation (CA). RFA and CA typically require general anaesthesia (GA) to allow the insertion of catheters, making them unsuitable for less fit patients. There is a substantial unmet clinical need for a non‐invasive treatment option for patients where surgery is not possible. RFA and CA have poor control rates in tumours larger than 3–4 cm and those that are centrally located and have a high risk of complications in tumours near the hilum.^2^, ^3^ Consequently, for many patients, although they have localised disease, the current standard of care is watchful waiting until the development of the metastatic disease. Metastatic disease is incurable, and treatment may be poorly tolerated in older patients with co‐morbidities. A prospective interventional clinical trial published by Siva et al. showed that a non‐invasive treatment option, stereotactic ablative radiotherapy (SABR) for primary RCC, was feasible and well tolerated.^4^ The treatment was shown to provide freedom from local progression, distant progression and overall survival rates at 2 years of 100%, 89% and 92%, respectively.^4^

In this prospective case series, we present our experience from a single institution of treating localised RCCs using CyberKnife and Linear Accelerator platforms including early patient outcomes and initial toxicity. The cohort was used to assess the feasibility of the delivery of SABR using CyberKnife and Linear Accelerator platforms.

## 2 MATERIALS AND METHODS

Patients were identified at a single institution by a urology multidisciplinary team (MDT) with the following eligibility criteria: presence of biopsy‐proven RCC of less than 6 cm maximum diameter, non‐surgical candidates and consensus at the urology MDT that radical treatment was appropriate. The decision to treat the RCC with SABR was because the patient was not fit for the GA needed for surgery or CA/RFA or was unsuitable for CA/RFA due to the location of the tumour. Majority of the patients who had >4 cm tumour had period of surveillance confirming progressive disease.

Although SABR technique can be used for tumours larger than 6 cm, for the purpose of keeping the cohort similar, we chose to keep maximum tumour size up to 6 cm.

Exclusion criteria included metastatic disease and factors precluding abdominal radiotherapy such as inflammatory bowel disease. Patients were subsequently discussed at the SABR MDT to confirm they were technically treatable and to decide on treatment platform.

Patients were treated as part of a prospective service evaluation with approval from the Novel Therapeutics Committee with peer‐reviewed approval of the protocol and funding from the hospital charity.

After patients gave their informed consent, the following data were collected: baseline Charlson co‐morbidity index, quality of life (QOL) assessment using Equation 5D and EORTC QLQ‐30 questionnaires, estimated glomerular filtration rate (eGFR), CT thorax, abdomen and pelvis (CT‐TAP) and dimercaptosuccinic acid (DMSA) split renal function, where clinically appropriate.

Face‐to‐face or telephone follow‐up data were collected at 2 weeks, 6 weeks, 3 months and 6 months, then 6 monthly thereafter. CT‐TAP (with intravenous [IV] contrast where appropriate) was performed post treatment at 6, 12 and 24 months. Toxicity using CTCAE V4.0 and QOL assessments were collected at 6 weeks, 3 months and 6 months, then 6 monthly thereafter for 3‐5 years. Some imaging was delayed during the coronavirus disease 2019 (COVID‐19) pandemic.

The primary endpoint was local control at 12 months and the secondary endpoints were safety, QOL, maintenance of renal function, response rate and overall survival.

### 2.1 Radiotherapy planning and motion management

The decision on treatment platform and technique, standard Linear Accelerator, using a volumetric modulated arc therapy (VMAT) technique, or on CyberKnife, was made at the SABR MDT. Patients treated on CyberKnife would require fiducial placement prior to treatment and those treated with a VMAT technique were reviewed for the use of abdominal compression. The choice on which platform to treat on took into account patient's ability to go through the minimally invasive procedure of fiducial insertion, under local anaesthetic, and tumour position, considering the proximity to organs at risk (OARs). For patients treated with VMAT, the decision to use abdominal compression was determined by the magnitude of tumour motion on a 4DCT (four‐dimensional computed tomography) assessment scan and on how close the OARs were. If motion did not result in the treatment target volume overlapping with OARs and compression would result in OARs closer to the target, then abdominal compression was not used. For patients where the kidney motion resulted in target overlap with OARs and CyberKnife was not an option, abdominal compression was used. CyberKnife platform was chosen for mainly lower pole tumours with proximity to the bowel due to the advantage of intra‐fraction tumour tracking.

For patients treated with CyberKnife, fiducials were inserted under local anaesthesia and CT guidance by a trained radiologist. Two sets of gold fiducials (two fiducials in each needle) were inserted with a separation distance of 2 cm. Fiducial placement occurred at least 10 days prior to planning CT scans to mitigate the effect of migration. Patients were scanned in a supine position in an inhale and exhale breath‐hold with 1‐mm scan thickness. For the exhale breath‐hold scan, IV contrast was given and this scan was used as the primary dataset.

For the VMAT treatments, patients were scanned with IV contrast in exhale breath‐hold, using a slice thickness of 2 mm, followed by a 4DCT scan to capture the extent of tumour motion. Patients were scanned in a supine position and immobilised using a vac bag, knee fix and with their arms above the head on a wing board. Abdominal compression was used for further immobilisation where appropriate.

### 2.2 Definition of target volumes and OARs

For patients treated with the VMAT technique, the 3D exhale breath‐hold scan was used as the primary scan for contouring and planning. The fused 4DCT was used to generate an ITV (interval target volume) using the 4D scan. A 5‐mm margin was then added in all directions to the ITV to create the PTV (planning target volume). For CyberKnife treatments, the exhale‐phase CT scan was used for contouring and identifying fiducial position. The CTV (clinical target volume) was the same as GTV (gross tumour volume) and a 3‐mm margin was added in all directions to create the PTV.

Small bowel, large bowel, duodenum, stomach, liver, spleen, contralateral kidney and spinal cord were outlined as OARs on the primary planning scan. Outlining volumes were reviewed by two clinical oncologists with SABR and uro‐oncology experience.

### 2.3 Dose fractionation

A dose of 26 Gy in one fraction or 42 Gy in three fractions, on alternate days, was prescribed. Patients with tumours > 5 cm or those that were in proximity to OARs were treated with three fractions. Treatment plans were produced with the aim to prescribe 100% of the prescription dose to at least 95% of PTV. For PTVs in proximity or overlapping OARs, a PTV_prescribe volume was created to ensure mandatory OAR constraints were achieved; the treatment dose was then prescribed to this structure.

### 2.4 Treatment planning

For CyberKnife patients, treatment plans were calculated on Precision 2.0.0.1 (Accuray Inc, Sunnyvale, CA, USA) with a ray‐tracing algorithm and a 1‐mm dose grid and using the IRIS variable aperture collimator. VMAT plans were created using RayStation 6.0.0.24 (RaySearch Laboratories AB, Stockholm, Sweden) using 6FFF beams with 1–2 arcs and, where possible, 180° arcs avoiding entrance through the contralateral side. A 2‐mm dose grid was used and plans were calculated using a collapsed cone algorithm.

Plans were created to meet the mandatory OAR constraints listed in Table 1. The dose constraints were amalgamated and modified based on constraints taken from the UK SABR Consortium guidelines,^5^ TG101 report^6^ and the FASTRACK (Focal Ablative STereotactic Radiosurgery for Cancers of the Kidney) trial.^7^ Conformity indices, as defined in the UK SABR Consortium guidelines,^5^ were also used.

**TABLE 1:** Planning dose constraints

Organ at risk	Constraint	
	#1	#3
PRV (planning risk volume) spinal canal	D0.1 cc < 12 Gy^7^	D0.1 cc < 18 Gy^7^
Small bowel	D0.5 cc < 26 Gy^7^ D5 cc < 22.5 Gy^7^ ≤12.5 Gy to full circumference of the bowel wall^7^	D0.5 cc < 30 Gy^7^ D30 cc < 12.5 Gy ≤12.5 Gy to full circumference of the bowel wall^7^
Duodenum	D0.5 cc < 12.4 Gy^6^ D5 cc < 11.2 Gy^6^ D10 cc < 9 Gy^6^	D0.5 cc < 22.2 Gy^5^ D5 cc < 16.5 Gy^5^ D10 cc < 11.4 Gy^5^
Stomach	D1.5 cc < 15.4 Gy^7^ D5 cc ≤ 22.5 Gy^7^	D0.5 cc < 30 Gy^7^ D5 cc ≤ 22.5 Gy^7^ D10 cc < 16.5 Gy^5^
Large bowel/colon	ALARA, D1.5 cc < 26 Gy^7^	ALARA, 1.5 cc < 42 Gy^7^
Liver	‐	At least 700 cc < 15 Gy^7^
Kidney (parenchyma)—ITV ipsilateral	ALARA—minimise volume of high dose to regions outside the ITV^7^	ALARA—minimise volume of high dose to regions outside the ITV^7^ V10 Gy < 33%^7^
Kidney (parenchyma) contralateral	V10 Gy < 33%^7^	V18.6 Gy < 33%^6^
Ureter	Record only 0.5 cc	D0.5 cc < 40 Gy^5^
Skin (5 mm rind)	D0.5 cc < 26 Gy^6^ D1.5 cc < 18 Gy^7^	D1.5 cc < 30 Gy^7^
Spleen	Record mean dose Record D0.5 cc	Record mean dose Record D0.5 cc

Abbreviations: ALARA, as low as reasonably achievable; ITV, interval target volume; PRV, planning risk volume.

### 2.5 Treatment delivery and verification

CyberKnife patients were treated on a CyberKnife VSI utilising synchrony motion tracking, with synchrony tracking uncertainties verified to be <1 mm. VMAT plans were delivered on the Elekta Versa machines with XVI images taken pre‐ and mid‐way through the treatment. Corrections were made for set‐up errors > 2 mm.

### 2.6 Dosimetric analysis

Analysis of dosimetric data and delineated structures was performed using in‐house analysis software and Python 3.6. Doses to critical structures were assessed against the constraints in Table 1. Target doses were assessed using the PTV D95% and D2% as well as the GTV mean dose.

BED (biologically effective dose) values for the tumour were calculated using the α/β ratios 2.6 and 6.9 Gy from a study of two cell lines.^8^ These relatively low values for the α/β ratio are a key motivation for SABR, although the absolute values of α and β are as small as may be expected for a highly radio‐resistant tumour.

### 2.7 Statistical analysis

Statistical analyses were carried out using Stata Version 17.0. Descriptive statistics, such as medians and interquartile ranges (IQRs) for continuous variables and frequency and percentages for categorical data, were used to summarise patient characteristics and their treatment. Tumour response was assessed using the RECIST (Response Evaluation Criteria in Solid Tumours) criteria for those with measurements available at the required time point.

The time‐to‐event outcomes of time to local control failure and overall survival were calculated from the date of the first SABR treatment until the date of local control failure, or death, respectively, or censored at the last follow‐up date to be alive and event free. These were summarised using Kaplan–Meier methods. Frequency of adverse events and their grade were summarised to assess safety.

### 2.8 Relationship between change in eGFR and the delivered dose

In addition, we explored the relationship between the change in eGFR and the delivered dose. To explore this relationship, the DVH (dose volume histogram) for healthy kidney (Kidney‐GTV) was converted to BED^9^:

$$\mathrm{BED}=\mathrm{nd}\left(1+\frac{d}{3}\right)$$

where *n* is the number of fractions, *d* is the dose per fraction and the α/β ratio was set to 3 Gy for a late effect. The generalised EUD (equivalent uniform dose) was used to condense the DVH into a single number. Using a volume parameter of 1, the correlation between the gEUD and mean change in eGFR post treatment was calculated.

$$\text{gEUD}={\left(\sum_{n=0}^{\text{Nbins}}v_{n}{\mathrm{BED}}_{n}^{a}\right)}^{\frac{1}{a}}$$

## 3 RESULTS

In total, 19 patients with primary RCC have successfully been treated with SABR over a 36‐month period. Patient characteristics and SABR treatment details are presented in Table 2. All patients had biopsy‐proven RCC with high Charlson co‐morbidity score^10^ reflective of co‐morbidity burden. The median follow‐up time was 17 months (range 5–32 months). Of the 19 treated patients who fulfilled predefined eligibility criteria, one patient had two RCCs in the same kidney. The median age was 76 years (IQR 64–82 years) and 47.4% were males.

**TABLE 2:** Patient and radiotherapy characteristics

Patient characteristics	Frequency, *n* (%)
**Number of patients with biopsy‐confirmed RCC**	19
**Tumour size in the largest dimension (cm)**	
Median (IQR)	4.5 (3.8–5.2)
Tumour size < 4 cm	5 (26.3)
Tumour size ≥ 4 cm	14 (73.7)
**Median age (IQR)**	76 (64–82)
**Sex**	
Male	9 (47.4)
Female	10 (52.6)
**Baseline eGFR**	
Median (IQR)	62 (43–74)
eGFR ≥ 90	3 (15.8)
eGFR 60–89	8 (42.1)
eGFR 30–59	7 (36.8)
eGFR 15–29	1 (5.3)
eGFR < 15	0
**Laterality**	
Left	10 (52.6)
Right	9 (47.4)
**Location**	
Upper pole	7 (36.8)
Middle pole	5 (26.3)
Lower pole	6 (31.6)
Middle and lower poles	1 (5.3)
**Charlson co‐morbidity score**	
11	1 (5.3)
9	5 (26.3)
8	4 (21.0)
7	1 (5.3)
6	4 (21.0)
5	4 (21.0)

Radiotherapy characteristics	Frequency, *n* (%)
**Prescribed fractions**	
1 fraction	8 (42.1)
3 fractions	11 (57.9)
**Platform**	
CyberKnife	8 (42.1) (#1 prescribed *n* = 5)
VMAT	11 (57.9) (#1 prescribed *n* = 3)
**Abdominal compression (VMAT only)**	
Yes	5 (45.5)
No	6 (54.5)
**PTV_prescribe**	
Number requiring PTV_prescribe volume	7 (63.2)

Abbreviations: eGFR, estimated glomerular filtration rate; IQR, interquartile range; PTV, planning target volume; RCC, renal cell carcinoma; VMAT, volumetric modulated arc therapy.

Eight patients were treated on CyberKnife and 11 using VMAT. Of the eight patients treated on the CyberKnife platform, five patients were treated with the 26 Gy in #1 regimen, with three patients treated with this fractionation using VMAT. Five of the 11 VMAT patients were treated in abdominal compression and seven of the 19 patients needed a PTV_prescribe volume due to proximity to the OARs. No PTV_prescribe was needed for the patients treated on the CyberKnife platform or one‐fraction treatments.

For single‐fraction cases and three‐fraction cases, the PTV D95% was a median of 26.1 Gy (range 25.9–27.5) and 40.7 Gy (range 20.0–42.1 Gy), respectively. Mean GTV doses ranged from 29.4 to 34.4 Gy with median of 31.9 Gy for the single‐fraction cases and 48.2 to 52.8 Gy with a median of 50.6 Gy for the three‐fraction cases.

**FIGURE 1:**
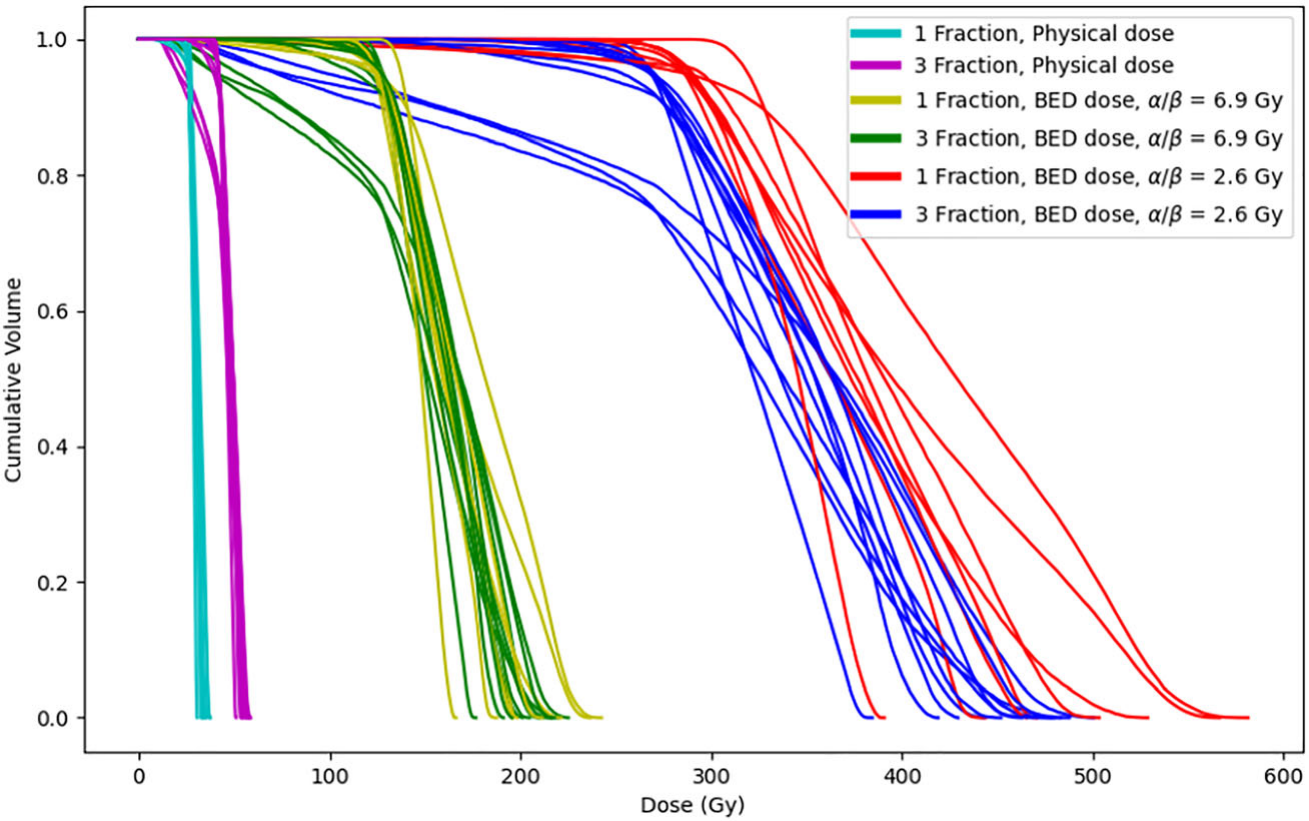
Planning target volume dose volume histograms for different values of the alpha–beta ratio. BED, biologically effective dose

BED‐corrected PTV DVHs are shown in Figure 1. For both assumed α/β ratios, there is minimal differentiation between one‐ and three‐fraction regimes, indicating that they are broadly iso‐effective.

The largest diameter of tumour (in cm) was measured at 6‐monthly intervals as an indication of tumour response. Tumour response could be assessed at 6 months for 15 patients who had CT scans at both baseline and 6 months and at 12 months for 14 patients with both baseline and 12 months of measurements available. Tumour response could not be calculated at 6 months for the four patients who did not have a scan at 6 months and for the five patients who did not have the scan at 12 months.

This was due to the deaths prior to the time point or being unable to book the CT scan due to the COVID‐19 pandemic. At 6 months, 3 patients had a partial response and 12 patients had stable disease. The median percentage reduction in tumour size from baseline to 6 months was 16.67% (IQR 2.7–28.0). At 12 months, five patients had a partial response and nine patients had stable disease. The median percentage reduction in tumour size from baseline to 12 months was 26.5% (IQR 7.9–34.5).

**FIGURE 2:**
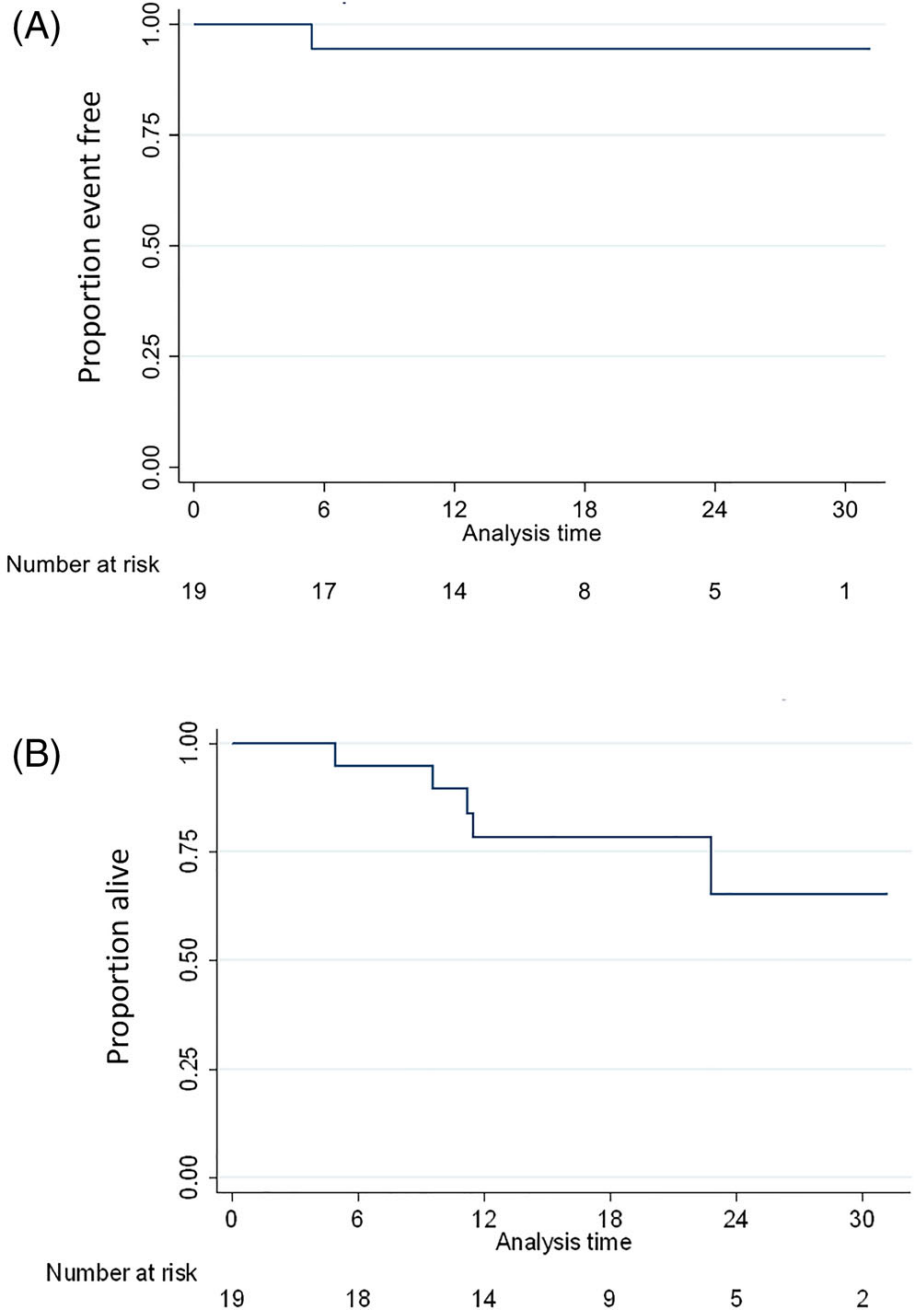
Kaplan–Meier for (A) time to local control failure and (B) overall survival

One patient had local progression and two patients had metastatic disease during the follow‐up period. The overall rate of local control at 6 and 12 months was the same: 94.4% (95% confidence interval [CI]: 66.6% to 99.2%; Figure 2A).

The overall survival rate at 6 months was 94.7% (95% CI: 68.1% to 99.2%) and the overall survival at 12 months was 78.3% (95% CI: 51.9% to 91.3%; Figure 2B). Seven patients died in total, of which six patients died of other causes not related to SABR or primary RCC (cardiac arrest, sepsis, heart failure or left ventricular failure; and two patients died of pneumonia).

The mean change in eGFR measurements from baseline to 6 months was −5.4 ml/min (95% CI: −10.3 to −0.6) and that from 6 to 12 months was −5.1 ml/min (95% CI: −9.0 to −1.1). The overall mean change from baseline to 12 months was −8.7 ml/min (95% CI: −15.3 to −2.1).

**FIGURE 3:**
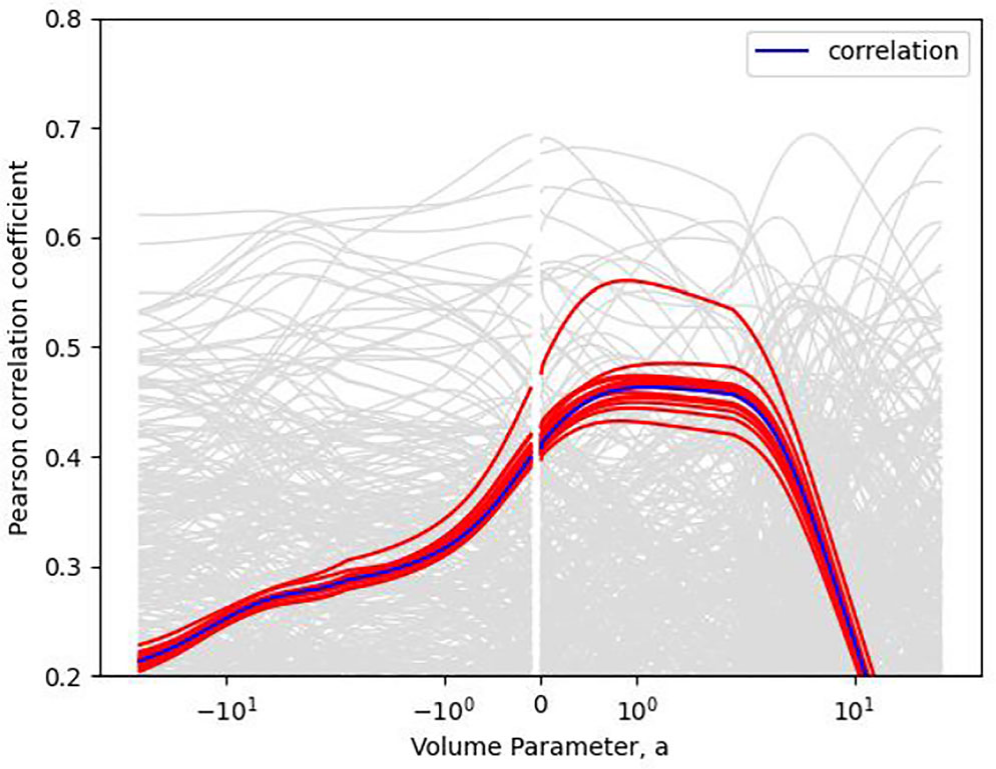
Correlation between biologically effective dose and estimated glomerular filtration rate as a function of volume parameter. The blue line shows the function over all the data; the red lines are the result of a leave‐one‐out analysis. The grey lines show results from random permutations of the results.

A related concern is the magnitude of any relationship between the change in eGFR and the delivered radiation dose. Using a volume parameter of 1, the correlation between the gEUD and mean change in eGFR post treatment was 0.46 and was borderline statistically significant (*p* = 0.05). Setting the volume parameter equal to 1 is based on the assumption that the kidney is a parallel organ. Figure 3 shows the correlation as a function parameter. The blue line shows the function over all the data; the red lines are the result of a leave‐one‐out analysis. A leave‐one‐out analysis was performed to identify the effect of individual cases on the shape of the curve. The statistical significance of this is not calculated although the light grey lines show the outcome of 10 000 permutations of the data.

Treatment toxicities are summarised in Table 3, showing the worst grade of each adverse event experienced by every patient during their follow‐up period. Only three patients experienced a serious adverse event of Grade 3 (colitis and haematuria at 6 weeks and anaemia at 9 months), which were resolved with conservative treatment. Fatigue was the most experienced adverse event with 3 (15.8%) patients experiencing Grade 2 and 12 (63.2%) patients had Grade 1.

**TABLE 3:** Worst grade of adverse event over each patient's follow‐up period

	Grade, *n* (%)				
	0	1	2	3	4
Fatigue	4 (21.1)	12 (63.2)	3 (15.8)	0	0
Gastritis	17 (89.5)	1 (5.3)	1 (5.3)	0	0
Anaemia	17 (89.5)	0	1 (5.3)	1 (5.3)	0
Pain	18 (94.7)	1 (5.3)	0	0	0
Nausea	14 (73.7)	1 (5.3)	4 (21.1)	0	0
Vomiting	17 (89.5)	1 (5.3)	1 (5.3)	0	0
Colitis	18 (94.7)	0	0	1 (5.3)	0
Diarrhoea	17 (89.5)	2 (10.5)	0	0	0
Haematuria	18 (94.7)	0	0	1 (5.3)	0
Haemorrhage	18 (94.7)	1 (5.3)	0	0	0

## 4 DISCUSSION

There has been increasing interest in the delivery of SABR to primary RCC in the United Kingdom after publication of a feasibility study of 37 patients by Siva et al. in 2017 as well as systematic review and meta‐analysis published in 2019.^4^, ^11^ After successful rollout of the SABR Commissioning through Evaluation programme from NHS England to other treatment sites, it is now becoming a well‐established technique within the United Kingdom. We eagerly await the results of the phase II study (FASTRACK II) by Siva's group.^7^

Here, we present the first UK cohort of patients with primary RCC receiving SABR with prospective follow‐up. Also described is the treatment and planning technique used for both CyberKnife and standard Linear Accelerator platforms.

Our early results show that SABR for primary RCC is feasible and can successfully be planned using two fractionation approaches, one and three fractions, on both treatment platforms. Furthermore, the majority (74%; 14/19) of tumours were above 4 cm, which is the group for whom other ablative treatments are less effective. Early toxicity and local control are promising, but further follow‐up is needed. Our results are consistent with the previously published data showing excellent local control with minimal toxicity.^4^, ^11^, ^12^, ^13^, ^14^ However, doses were inconsistent in these studies, and we have aimed to align treatment planning and doses with the largest prospective cohort.^4^

In our cohort, only one patient had a solitary kidney with a large RCC. Due to location and size of the tumour, it was felt that CA was not appropriate. Nephrectomy would have deemed the patient anephric, requiring unavoidable dialysis, an option that the patient refused. The option of SABR has had significant impact on the QOL for this patient as well as saving of the cost of the dialysis for the NHS. Demonstrating SABR would be a promising non‐invasive treatment especially for solitary kidneys.^15^

Radiological response assessment following SABR has been challenging especially for renal cancers but has not been unique.^4^, ^16^, ^17^, ^18^, ^19^ It is well known that tumour size can increase initially post SABR with subsequent reduction over years.

For this reason, we did not image patients at 3 months due to known pseudo progression seen post treatment. Our patient cohort showed that local control at 6 months was 94.4%, and at 12 months, local control was maintained. The overall survival rate was 94.7% (95% CI: 68.1% to 99.2%) at 6 months and 78.3% (95% CI: 51.9% to 91.3%) at 12 months.

In addition, post‐radiotherapy biopsy results can also be unreliable. Post brachytherapy for prostate cancer, biopsies can be positive for tumour cells for up to 2 years but do not correlate to biochemical disease control.^20^ One of the dose escalation studies of renal SABR showed one of the positive biopsies at later follow‐up turned negative without any further treatment.^13^

A large meta‐analysis of 99 retrospective studies published by Kunkle et al. showed local tumour progression rates of 5.2% after CA and 12.9% after RFA.^21^ The mean tumour size of the cohort was much smaller (24.6 mm) as compared with our cohort. A total of 79.7% lesions had pathologically confirmed RCC, 12.2% were benign and 8.1% had unknown or indeterminate pathological findings.^21^ In our cohort, all patients had biopsy‐confirmed RCC.

The advantage of renal SABR is not only to offer the option of non‐invasive ablation (VMAT technique) but also to conserve renal function. In the short follow‐up, our data show that, although there is an initial drop in renal function, overall, the majority of the function was maintained. No patient has needed dialysis.

There is a well‐established link between lower GFR and increased risk of death from cardiovascular events.^22^ A previous publication of SABR for primary RCC has shown impressive preservation of renal function with mean decrease in eGFR of 5.5 ml/min, corresponding to rise in serum creatinine of 28.1 μmol/L,^23^ and similar to the mean decrease at 6 months of 5.4 ml/min that was seen in our study. In the prospective trial published by Siva et al., at 1 year, eGFR was reduced by 11 ml/min (95% CI: 6 to 17).^4^ These results, although encouraging, need longer follow‐up for confirmation of renal function preservation.

There is a paucity of literature on the response of healthy kidney to partial irradiation, which was discussed in the QUANTEC (Quantitative Analysis of Normal Tissue Effects in the Clinic) article on kidney toxicity.^24^ A commonly used value of the volume parameter is 0.7, which dates to Emami et al.^25^ In this case, we have a highly heterogeneous kidney dose with small volumes receiving the treatment dose with the contralateral kidney limited to 10 Gy to 33% of the volume or 18.6 Gy to 33% for one and three fractions, respectively. Figure 3 illustrates how the correlation between BED and eGFR is affected by the assumption of volume parameter. For small values of the volume parameter, the correlation is between 0.2 and 0.4, rising to a maximum of 0.46 for volume parameters around 1–5, before falling rapidly. It seems highly unlikely that a naturally parallel organ such as the kidney would have a volume parameter much greater than 3.

The majority of toxicity in our cohort was Grade 1 and most common being fatigue. A large series of renal tumours treated with CA recently showed that major complication rate (Clavien–Dindo classification ≥ Grade 3) was 2.2% with immediate procedural complication rate of 5.9%.^26^ In this series, mean tumour diameter was 28.5 mm. The systematic review by Correa et al. reported 1.5% Grade 3–4 toxicity with SABR.^11^ Our cohort showed that treatment is well tolerated with no Grade 4 toxicity. Three patients had Grade 3 toxicity (colitis, haematuria and anaemia), which were resolved with conservative management. Our low toxicity rate is in keeping with other publications of SABR for primary RCC.^11^, ^13^, ^27^ There was no treatment‐related mortality. Data on QOL have also been collected and will be published with additional late toxicity information after a further follow‐up period.

The OAR constraints used for this cohort have been adapted and combined from various sources.^5^, ^6^, ^7^ The use of different definitions of maximum dose to a structure and different volume parameters within the publications has made the process challenging and it is acknowledged that further work needs to be carried out. The allowed values for stomach and bowel were higher than routinely used in the United Kingdom. However, in this cohort, there were only two patient plans that exceeded the UK consensus^5^: one in the case of large bowel, where 0.5 cc = 42.9 Gy, and one for small bowel, where 5 cc = 21.4 Gy (UK SABR Consortium constraints for these parameters are 28.2 and 17.7 Gy, respectively). Neither of these patients reported more than Grade 2 toxicity.

Our prospective cohort has limitations, including single‐institutional study, small sample size and short follow‐up. In time with longer follow‐up, we hope to update our results.

Although we recognise that, in this cohort, 37% of patients died, none of them died either due to the treatment received or due to treatment‐related toxicity or metastatic disease, giving hope that if confirmed in future studies with longer follow‐up, this treatment may be an option for patients who do not have surgical option and have less co‐morbidities than reflected in this cohort.

There is an urgent need for a wider collection of prospective data to firmly establish this treatment technique. With an aging population and increased detection of the renal cancers, there is an unmet need for non‐invasive effective treatment for primary RCC.

## 5 CONCLUSION

Our study has demonstrated that SABR for RCC is safe, feasible and deliverable in a UK institution with previous SABR experience, using both one‐ and three‐fraction regimes. It is our hope that this information will contribute to the development of a multicentre clinical trial or national guideline to be used in the UK clinical oncology community.

## DISCLOSURE OF INTEREST

None.

## AUTHOR CONTRIBUTIONS

Dr Anjali Zarkar and Dr Dan Henderson: concept, protocol development and novel therapies application. Dr Anjali Zarkar and Helen Howard: protocol development, writing, discussion of the results and proof reading. Antony Carver: analysis of the correlation between BED and eGFR. Helen Howard and Geoff Heys: physics input in the protocol development. Anjali Zarkar, Julie Kilkenny and Helen Howard: motion management of the protocol development. Victoria Harrop and Sarah Tutill: prospective data collection. Andrea Marshall and Nada Elbeltagi: statistical analysis.
